# Towards standardized gut microbiota diagnostics: normobiosis beyond geographical borders

**DOI:** 10.1080/19490976.2026.2701485

**Published:** 2026-07-12

**Authors:** Pranvera Hiseni, Kari Furu, Vivian Wang Pedersen, Lise Øverland, Graceline Tina Kirubakaran, Christina Casén

**Affiliations:** a Genetic Analysis AS, Oslo, Norway

**Keywords:** Dysbiosis, gut microbiota, normobiosis, standardization, microbiome profiling, clinical biomarkers

## Abstract

Defining clinically meaningful reference states of the human gut microbiota remains a major barrier to the clinical translation of microbiome testing, largely due to variability across populations. We aimed at evaluating whether dysbiosis can be identified in a standardized, geography-independent manner, using a composite, system-level microbiome diagnostic framework. We performed a retrospective observational analysis of 831 adult stool samples collected from healthy individuals and patients with inflammatory bowel disease, irritable bowel syndrome, or other chronic inflammatory conditions across seven countries. In addition, U.S. National Institute of Standards Technology (NIST) human fecal reference materials were analyzed. Dysbiosis was assessed using a fixed microbial marker panel and composite distance metrics anchored to a clinically validated healthy Scandinavian reference population, using GA-map® Dysbiosis Test as an exemplar of this diagnostic framework. The diagnostic framework reproducibly identified normobiosis and dysbiosis across geographically distinct populations (USA, Canada, Germany, Italy, and the UK). Severe dysbiosis was detected with high specificity (93.6%) and positive predictive value (90.2%), independent of subjects' country of origin. Healthy individuals showed highly comparable dysbiosis index distributions across regions. These findings demonstrate that clinically useful dysbiosis diagnostics do not require geographically tailored reference populations and support the feasibility of standardized, geography-independent microbiome diagnostics for clinical application.

## Introduction

Defining a clinically actionable reference state of the human gut microbiota (“normobiosis”) remains one of the most persistent challenges in translating microbiome research into routine clinical practice. This represents a conundrum at a time when associations between gut microbiome imbalances (“dysbiosis”) and human health have become cemented.

The composition of gut microbiota in healthy populations across the world is confounded by various factors, including traditional cuisine, lifestyle, and environmental exposures.^1^ In addition, the composition exhibits high intra-individual variability, reflecting its responsiveness to daily fluctuations in diet, sleep, medication use, bowel habits, and physical activity.^2‐4^ As a result, uncertainty persists regarding how microbiome data should be interpreted and reported in routine medical screening globally,^1^ ^,^ ^5^ despite their considerable potential to play a central role in personalized medicine.

Addressing the complexity of the matter, a recent international consensus statement on microbiome testing in clinical practice has declared that “*generally, there is not enough information to report strict healthy reference ranges of species relative abundance*”.^5^ The same also noted that “*there is insufficient evidence to include any dysbiosis index in the report of microbiome testing*”,^5^ further enforcing skepticism around the clinical utility of microbiome-based diagnostics.

While we agree with many concerns outlined in the aforementioned consensus statement,^5^ we underline that many of the addressed limitations arise from the assumption that microbiome diagnostics must rely on high-dimensional taxonomic profiles, rather than composite system-level deviations from a reference state.

We argue that a key limitation of high-dimensional microbiome datasets is their inherent susceptibility to noise accumulation and multiple testing burdens, which can compromise reproducibility and limit transferability across independent cohorts.^6^ ^,^ ^7^ In addition, the compositional nature of microbiome data introduces dependencies between taxa, complicating statistical interpretation and increasing susceptibility to spurious associations.^6^ As a result, high-dimensional models trained on one cohort may capture population-specific microbial patterns rather than generalizable signatures of health or disease. This can lead to reduced diagnostic performance and inconsistent interpretation when applied to geographically distinct populations.

In contrast, approaches based on predefined marker panels and system-level composite metrics reduce the dimensionality of the data and constrain the analysis to reproducible microbial signals. By anchoring deviations to a fixed reference model, such frameworks are less sensitive to cohort-specific variation and better suited for cross-population applications.

We highlight GA-map® Dysbiosis Test, a molecular diagnostic assay based on a fixed marker panel and an algorithm anchored in a clinically validated healthy reference Scandinavian population, as an example of a platform that addresses several challenges periodically emphasized by the scientific community.^5^ We provide evidence that the inclusion of a dysbiosis index in microbiome testing reports offers significant value for clinical monitoring and disease prevention, supporting its clinical applicability. Furthermore, we demonstrate that identification of dysbiosis by GA-map® Dysbiosis Test is not restricted to Scandinavian samples, highlighting the utility of the test beyond its original reference population.

### GA-map® Dysbiosis Test principle

GA-map® Dysbiosis Test reports a dysbiosis index (DI) of a sample on a scale from 1 to 5, where 1 and 2 are normobiotic, 3 is classified as mildly dysbiotic, and 4 and 5 represent severe dysbiosis.

In short, the classification of a sample is performed by computing the collective distance between the abundances of 48 commensal microbial markers compared to those obtained from a clinically validated healthy adult reference (Scandinavian) population.^8^ Certain markers represent single species (e.g., *Akkermansia muciniphila, Faecalibacterium prausnitzii)*, while others represent members of broader taxonomic levels (e.g., Pseudomonadota), providing the method with a uniquely comprehensive analytical scope and stability against daily intra-individual compositional fluctuations.

**Figure 1.**
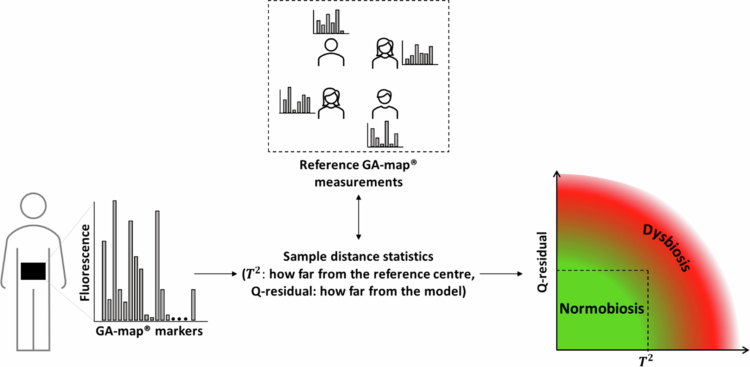
Schematic overview of a GA-map® Dysbiosis Test analysis workflow. Incoming fecal samples are analyzed for the presence and abundance of predefined forty-eight microbial markers. Resulting profiles are compared with those from a healthy reference population (>160 adults) to generate quantitative distance statistics. Samples with elevated statistics (T^2^ or Q-residuals passing normobiosis borders) are classified as dysbiotic.

While the positioning of a sample is directed by the abundance of each marker, the distance from the tested sample to the healthy reference is calculated in a multidimensional space applying fifteen principal components.^8^ This ensures that the classification of samples is not based on linear relationships between gut microorganisms (see Figure 1). In addition, independent algorithms compute the abundance of individual bacteria markers relative to the healthy reference (reduced, normal, elevated) and alpha diversity measure, serving as additional test features.

Data acquisition of the forty-eight markers is performed using a highly standardized multiplexed GA-map® assay, employing forty-eight separate oligonucleotides (“probes”) designed to hybridize to unique sequence signatures of 16S rRNA gene, each representing unique target microorganisms. Probes become labeled with a fluorophore only upon hybridization to their target sequence. The fluorophore signal is proportional to the abundance of the target, allowing direct and standardized signal interpretation. Extensive details related to method development and validation are described in Casén et al. (2015).^8^

While the method is based on targeting unique DNA sites, it does not rely on DNA sequencing techniques. This is an especially relevant point when considered alongside the recent international consensus statement on microbiome testing in clinical practice which concluded that “*appropriate methods for gut microbiome community profiling include amplicon sequencing and whole-genome sequencing*”.^5^ Such guidelines understandably prioritize sequencing-based methods for discovery; however, an exclusive reliance on sequencing-based approaches can pose challenges for standardization and reproducibility^9^ when translated into routine clinical diagnostics.

Through this work, we illustrate how diagnostic frameworks based on fixed marker panels, reproducible statistics, and invariant reference populations can enable clinically actionable microbiome interpretation across cohorts and time.

## Results and discussion

### GA-map® Dysbiosis Test classifies reference samples from healthy U.S. donors as “normobiotic”

To showcase the test's ability to correctly categorize human fecal samples beyond Scandinavian borders, the test was applied to a set of commercially available stool references representing healthy human adults: NIST (National Institute of Standards Technology) human fecal material collected from omnivores, and NIST human fecal material collected from vegetarians, both originating from the US.

The decision to test samples from NIST was deliberate, particularly in light of the findings by Servetas et al., who observed that identical NIST samples sent to seven different direct-to-consumer gut microbiome testing companies (all employing DNA sequencing methods) produced entirely different results.^9^ More concerningly, the results for a few sample replicates tested by the same provider yielded inconsistent outcomes.^9^ Beyond the technical challenges associated with DNA sequencing-based methods and the utility of the reported results, the study revealed that some of the providers utilized externally established healthy microbiome references to interpret results,^9^ raising concerns about reliability and clinical relevance. We argue that for clinical applicability, diagnostic frameworks benefit from employing the same reproducible methodology to establish healthy reference ranges and analyze patient samples, thereby supporting consistent interpretation of dysbiosis.

**Figure 2.**
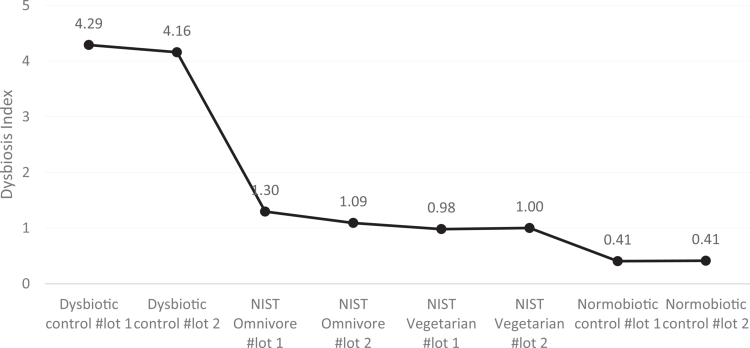
Dysbiosis index results for four different samples tested with two separate GA-map® Dysbiosis Test reagent kit lots. Dysbiotic and Normobiotic controls represent anonymized fecal samples used as test controls. For reporting purposes, decimal values are always rounded up to the nearest integer.

In contrast to the inconclusive results reported by Servetas et al., the GA-map® Dysbiosis Test identified normobiosis in both US reference samples. When analyzed using two independent GA-map® Dysbiosis Test kit lots over two separate runs, the NIST omnivore replicates yielded DI values of 1.3 and 1.09, whereas the NIST vegetarian sample yielded DI values of 0.98 and 1.00 (Figure 2). For reporting purposes, decimal values are always rounded up to the nearest integer (NIST omnivore resulting in DI 2, NIST vegetarian in DI 1).

Interestingly, the NIST sample collected by omnivores registered a notable reduction of the Bacteroidota phylum (Supplementary Figure S1). A decrease of these gut bacteria is often considered a marker for a diet depleted in fiber, high in saturated/trans fats and simple sugars - a signature of the Western diet.^10^

Such results highlight the complexity of microbiome data analyzes and illustrate the limitations of drawing conclusions based on isolated taxonomic changes. A complex system like the network of gut microorganisms is more than a mere sum of its parts. Despite the decrease in Bacteroidota (i.e., lower abundance compared to the reference range for this marker), the GA-map® Dysbiosis Test algorithm has classified the sample as being “normal”, recognizing the overall microbiota pattern rather than individual taxon deviations in the tested sample as such.

We acknowledge and emphasize the importance of identifying and reporting associations between individual microbial species and health indicators such as body mass index, glucose metabolism, and inflammation markers.^11^ However, accumulated evidence in the field demonstrates that no single marker is sufficient to reliably predict health outcomes. Instead, the use of a composite measure, such as dysbiosis index, represents a more robust, convenient, and informative approach for reporting results.

### Clinical utility of GA-map® Dysbiosis Test across different populations

**Figure 3.**
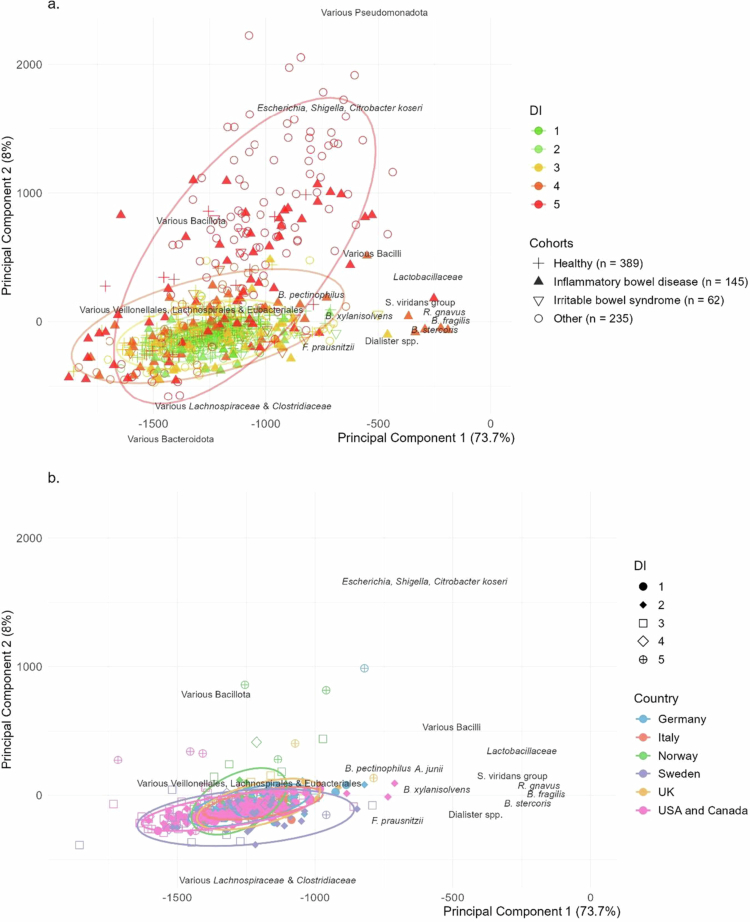
Principal component analysis plots based on normalized GA-map® Dysbiosis Test microbial marker signals. Each dot represents one sample. Panel a. includes samples from various health condition cohorts (indicated by shapes), colored based on the resulting dysbiosis index (DI). The first principal component accounts for most of the variation in the data (73.7%). The second principal component captures more extreme cases, primarily from individuals with inflammatory bowel disease and other chronic inflammatory diseases, which also corresponds to extreme DI scores. Panel b. focuses on the view of samples collected from healthy individuals only (389 in total), shaped based on their DI and colored based on country of origin. The samples do not show clustering and separation tendencies based on donors' origin. Loadings in both panels indicate taxa contributing most strongly to variation along the principal components.

A major barrier in adapting dysbiosis detection systems in clinical settings is their applicability across various parts of the world. To further demonstrate the utility of the GA-map® Dysbiosis Test across geographically distinct places, Figure 3 presents a principal component analysis (PCA) plot based on bacteria marker measurements derived from over 800 samples obtained from individuals with diverse health conditions across multiple countries. The results show that the separation of cohorts based on health status is well captured by the dysbiosis index algorithm, and that the algorithm is neither biased nor confounded by the sample country of origin.

The first principal component (73.7% variance) primarily reflects a gradient from commensal, butyrate producing taxa (e.g., *Lachnospiraceae* ^12^) towards profiles enriched in opportunistic and inflammation-associated bacteria (e.g., *Ruminococcus gnavus,* ^13^ *Bacteroides fragilis,* ^14^ *Streptococcus viridans* group.^15^) On the other hand, the second principal component captures 8% additional variation within dysbiotic profiles, particularly associated with expansion of pathobionts, particularly Pseudomonadota.^16^ This axis also juxtaposes Bacteroidota and Bacillota, with Bacillota standing in association with the dysbiotic profiles.

Inflammatory bowel disease (IBD), and samples collected from individuals suffering from other chronic inflammatory diseases or symptomatic non-IBD patients (labeled on the plot as “other”), separate best along the second principal component axis. Such a movement corresponds to higher DI scores, indicating that the DI reflects a system-level microbiome shift captured by the principal component structure.

Figure 3 (panel b) provides a filtered view of samples collected only from healthy individuals. None of the samples originating from different countries cluster separately along the first two principal components, which explain 81.7% of the variation in the data. Given that the GA-map® Dysbiosis Test algorithm is based on PCA statistics, the results demonstrate that the test is not influenced by country-specific bias, supporting its applicability across geographically diverse populations.

**Figure 4.**
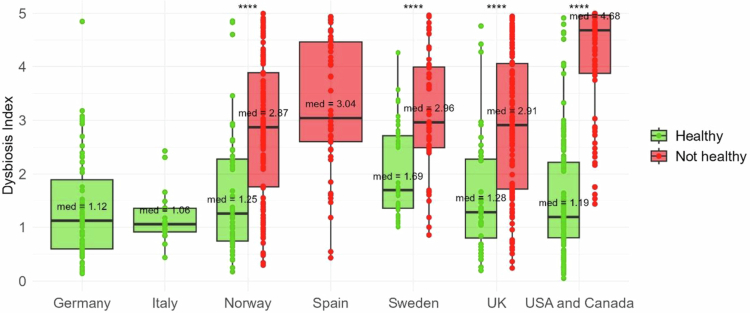
Boxplot with individual data points overlaid showing dysbiosis index (DI) distribution across countries stratified by health status, with median values (“med”) indicated for each group. The four stars at the top of the chart indicate the level of significance of the difference in DI between healthy and non-healthy cohorts within country (Wilcoxon test, *p* < 0.005).

To further assess geographic transferability, we compared DI distributions among healthy and not healthy individuals from Scandinavia and non-Scandinavian countries. Median DI values and the proportion of individuals classified as normobiotic were highly similar across cohorts, and no non-Scandinavian country showed a systematic increase in severe dysbiosis classifications for the healthy cohort (Figure 4).

Specifically, severe dysbiosis was observed in 4/67 (6%) German healthy individuals, 0/18 healthy Italian individuals, 4/52 (7.7%) healthy Norwegians, 5/36 (13.9%) healthy Swedish individuals, 3/40 (7,5%) UK individuals, and 9/176 (5.1%) US healthy individuals.

The decimal DI average of all healthy samples resulted in 1.53 (rounded up to DI 2, normobiotic). More importantly, the samples collected from IBD, irritable bowel syndrome (IBS), and patients suffering from other diseases, regardless of their origin, resulted in a decimal average DI of 3.19 (rounded up to DI 4, severely dysbiotic), highlighting the test's ability to separate disease cohorts from a healthy population, independent of geographic origin.

It is worth noting that DI distributions among the non-healthy cohorts were more variable across countries, likely reflecting the heterogeneity of the non-healthy population, which included differences in cohort composition across regions.

**Figure 5.**
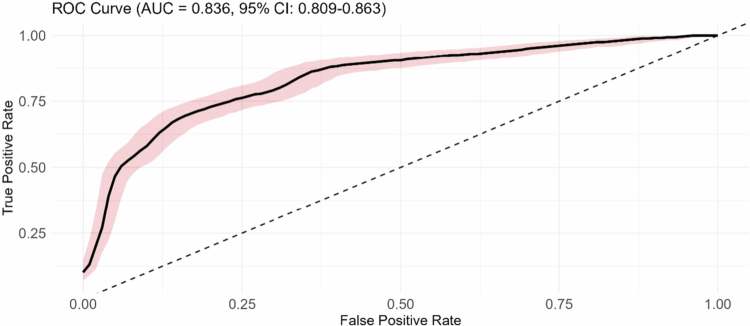
Receiver operating characteristic (ROC) analysis of the dysbiosis index (DI) for discrimination between healthy and not healthy individuals, with higher DI indicating a greater likelihood of being classified as not healthy. The solid line represents the empirical ROC curve, and the shaded area indicates the 95% confidence interval.

To further assess the discriminatory performance of the dysbiosis index across the combined cohort, we performed receiver operating characteristic (ROC) analysis comparing healthy and non-healthy individuals. The DI demonstrated good classification performance, with an area under curve (AUC) of 0.84 (Figure 5), indicating robust separation between health states across a heterogeneous multinational population.

Among all the tested samples, 255 resulted in severe dysbiosis (DI≥4). The majority of these samples (90.2%) were collected from individuals affected by various medical conditions, underlining the high positive predictive value of the test. Out of 389 healthy individuals in total, twenty-five resulted in severe dysbiosis, corresponding to a specificity of 93.6% when severe dysbiosis is considered a positive result.

Although the proportion of healthy individuals classified as severely dysbiotic was comparatively low (6.4%), this group warrants closer monitoring given their markedly different gut microbiome composition. This is particularly important in light of the findings demonstrating that various health conditions, including Parkinson's disease,^17^ the onset of celiac disease,^18^ and osteoarthritis,^19^ are preceded by dysbiosis.

In the present study, detailed metadata on environmental and lifestyle factors were not uniformly available across all cohorts, and therefore their potential confounding effects could not be fully assessed. Similarly, due to lack of complete metadata, stratified analyzes by disease subtype or medication for the non-healthy group were not performed. These factors are known to influence gut microbiota composition and may contribute to variability in DI distributions. However, the highly similar distributions of DI among healthy individuals across different countries suggest that the framework is robust to such variability. Nevertheless, we acknowledge that future studies incorporating detailed environmental and clinical metadata, in addition to a larger sample size are warranted to further evaluate potential confounding effects.

## Clinical relevance and supporting evidence

Previous studies using the GA-map® Dysbiosis Test have demonstrated its ability to identify disease-associated microbiota patterns and to distinguish between patient groups and healthy controls across diverse clinical contexts. For example, distinct microbiota signatures and elevated DI values have been reported in ankylosing spondylitis and linked to intestinal inflammation, as reflected by fecal calprotectin levels.^20^ Similarly, a large European multicenter study in IBD showed associations between microbiota composition, disease phenotype, and inflammatory markers, while also highlighting the complexity and heterogeneity of these conditions.^21^ Additional studies have demonstrated that DI is associated with disease activity and physical function independently of treatment and other confounders.^22^

The present study is cross-sectional and does not directly assess temporal dynamics or treatment response. However, previous longitudinal studies have shown that DI values can change following therapeutic interventions, such as fecal microbiota transplantation, and are associated with clinical improvement in IBS, supporting the potential utility of this framework for monitoring microbiome changes over time.^23^ Nevertheless, the use of the DI for longitudinal monitoring requires further validation in prospective studies.

The GA-map® Dysbiosis Test is currently intended for use in adult populations. However, recent large-scale analysis suggests that, despite age-related variation in individual microbial markers, overall DI values remain comparable across broader age ranges (2–70 y),^24^ supporting the concept that dysbiosis reflects a system-level state rather than isolated taxonomic shifts.

## Methodological scope, limitations, and complementary approaches

This study is based on a single, predefined diagnostic framework and does not include direct comparison with sequencing-based approaches such as 16S rRNA gene sequencing or shotgun metagenomics. While such comparisons are valuable for benchmarking different analytical strategies, the objective of this study was not to evaluate competing technologies, but rather to assess the performance and transferability of a standardized, clinically implemented microbiome diagnostic tool.

Notably, comparative analyzes between the GA-map® Dysbiosis Test and 16S rRNA gene sequencing approaches have been previously reported, demonstrating strong correlations in taxonomic signals and substantial concordance in dysbiosis classification across methods.^8^

While the present framework focuses on predefined taxonomic markers and a standardized reference-based model, alternative approaches to microbiome analysis offer complementary insights. Functional profiling methods, including shotgun metagenomics and metabolomics, provide information on microbial gene content and activity, and may therefore capture functional alterations not reflected in taxonomic composition alone.

Accordingly, GA-map® Dysbiosis Test is designed to identify deviations from a normobiotic reference state at the level of selected microbial markers and composite system-level patterns. As such, it does not directly measure microbial gene content, functional pathways, or metabolite production. Furthermore, although the method demonstrates robustness across populations, it is not specifically designed to detect subtle intra-individual fluctuations over short time scales.

These considerations highlight that different analytical frameworks capture distinct aspects of microbiome variation, and should be viewed as complementary rather than competing approaches.

## Conclusion

Inter- and intra-personal variations in the composition of human gut microbiota are extraordinarily high,^1^ making any precise definition of a “healthy” microbiome effectively unattainable. However, we show that by focusing on deviations from what is universally shared rather than on individual variability, we can move toward more reliable, clinically actionable definitions of microbiome imbalance.

The results presented here demonstrate that geographic variation in gut microbiota composition does not preclude the detection of dysbiosis when assessed using a standardized, reference-based framework. Rather than relying on compositional similarity of individual taxa, dysbiosis should be understood as a complex, system-level deviation from a normative state, captured through mathematical models like the one exemplified here.

Our findings also demonstrate that high taxonomic resolution is not a prerequisite for clinical utility, supporting a shift from purely descriptive microbiome analyzes towards standardized, metric-based frameworks.

Although the healthy reference population used in the predefined algorithm is Scandinavian, the observed stability of DI distributions across healthy cohorts from multiple non-Scandinavian countries supports transferability of the framework beyond the training population. Nevertheless, larger validation studies involving more ethnically diverse healthy populations will be important for further establishing global generalizability.

Overall, these results support the feasibility and clinical utility of standardized, low-dimensional microbiome diagnostic frameworks as reproducible and scalable tools for clinical assessment of gut microbiota across diverse populations.

## Method

### Laboratory analysis of the samples

All analyzes were performed at Genetic Analysis AS premises (Oslo, Norway). A total of 831 samples were analyzed using a standard GA-map® Dysbiosis Test Instructions for Use over the course of 12 y (2013–025). Among the samples, 389 were collected from healthy subjects, recruited in studies from Norway, Sweden, USA, Canada, Germany, Italy, and UK. The subjects were recruited and documented as healthy after being interviewed by health care personnel. Of the remaining, 145 were collected from subjects diagnosed with inflammatory bowel disease, 62 from individuals suffering from irritable bowel syndrome, and 235 from individuals suffering from other chronic inflammatory diseases. All results were anonymized and their use in this study is deemed secondary.

Following internal standard bioinformatic pipelines, all raw fluorescence readings were well-to-well, plate-to-plate, and batch-to-batch normalized to correct for technical variations between readings.

A detailed laboratory workflow is presented in the GA-map® Dysbiosis Test Lx v2 Instructions For Use (S8-044-A1 v12) (Supplementary file 1).

The two human fecal materials from NIST (RM8048), one from vegetarian donors and one from omnivores, were analyzed in accordance with S8-044-A1 v12, with the exception of an additional pre-processing step described below.

The RM8048 standard fecal materials were completely thawed on ice, followed by a thorough mix by vortexing to ensure sample homogeneity and even resuspension of the material.

The thawed material (100µl of each sample) was treated with 500µl eNAT™ buffer, to ensure uniform treatment of all samples processed with the standard GA-map® Dysbiosis Test. The samples were mixed by vortexing and incubated further for 30min at room temperature prior to entering step 1 of “Performing gDNA extraction” section in S8-044-A1 v12.

### Dysbiosis index calculations

The dysbiosis index (DI) is derived from a predefined algorithm based on principal component analysis (PCA) of probe-based microbiota profiles, as originally described in Casén et al. (2015).^8^ Briefly, a normobiotic reference model was constructed using a clinically validated healthy population, and deviations from this reference were quantified using Hotelling's $T^{2}$ and Q statistics. These metrics were combined into a single Euclidean distance measure, which was transformed into the DI score on a scale from 1 to 5. A threshold of ≥3 was defined as dysbiosis based on confidence limits derived during model calibration.

The model was developed using separate training and test datasets and validated in independent cohorts, demonstrating robustness and reproducibility. Full details of model development, threshold calibration, and technical validation are provided in the original publication.

## Supplementary Material

Supplementary Figure 1 Supplementary Figure 1

Supplementary material.pdf

## Data Availability

The datasets generated and/or analyzed during the current study are not publicly available due to contractual, ethical, and commercial restrictions, including protection of proprietary assay details and de-identified human subject data. Access to de-identified data may be granted upon reasonable request and with appropriate data use agreements. Requests for access should be directed to the corresponding author (email: [ph@genetic-analysis.com]) and will be considered in accordance with institutional policies.

## Use of AI statement

The authors confirm that they have read and comply with the Taylor & Francis AI Policy. An AI-assisted tool (OpenAI, ChatGPT 5.2) was used to support language editing, formatting, and improvement of clarity and structure of the manuscript text. The tool was also used to generate ideas on how to improve manuscript based on original data, assist in drafting and refining responses to reviewers, in addition to being used for literature searches and Rstudio coding assistance. All work was performed under strict and rigorous human revision. No AI tools were used to generate original scientific data or influence the scientific conclusions of the study.
