# Supplementary material for: Towards standardized gut microbiota diagnostics: normobiosis beyond geographical borders
Source: Gut Microbes. 2026 Jul 12;18(1):2701485. doi: 10.1080/19490976.2026.2701485 (PMC13367077; doi:10.1080/19490976.2026.2701485)
Supplement: Supplementary Figure 1 [file KGMI_A_2701485_SM6067.docx]

## Supplementary material

Figure S1. Bacteria relative abundances for four different samples tested with GA-map® Dysbiosis Test over two separate runs, utilizing separate reagent kit lots. Dysbiotic and Normobiotic controls represent anonymized fecal samples used as test controls. The coloring of the tiles highlights the deviations from the norm (0), red tiles indicating severe deviations (±3 deviations). The abundance is considered elevated if the score > 0 and reduced if the score < 0. The deviations are reported on a scale from –3 to +3. The results identity between runs was calculated by subtracting the percentage of markers with non-identical results from 100%.
