# Supplementary material for: Towards standardized gut microbiota diagnostics: normobiosis beyond geographical borders
Source: Gut Microbes. 2026 Jul 12;18(1):2701485. doi: 10.1080/19490976.2026.2701485 (PMC13367077; doi:10.1080/19490976.2026.2701485)
Supplement: Supplementary material.pdf [file KGMI_A_2701485_SM5833.pdf]

GA-map® | Dysbiosis Test Lx v2

INSTRUCTIONS FOR USE (IFU)

Dysbiosis Test Lx v2 – 96 Tests

REF 1001

Revision 12

CE

IVD

Genetic Analysis

Standard terms and conditions for use of assay product

By opening the packaging containing this Assay Product (which contains fluorescently labeled microsphere beads authorized by Luminex Corporation) or using this Assay Product in any manner, you are consenting and agreeing to be bound by the following terms and conditions. You are also agreeing that the following terms and conditions constitute a legally valid and binding contract that is enforceable against you. If you do not agree to all of the terms and conditions set forth below, you must promptly return this Assay Product for a full refund prior to using it in any manner.

You, the customer, acquire the right under Luminex Corporation's patent rights, if any, to use this Assay Product or any portion of this Assay Product, including without limitation the microsphere beads contained herein, only with Luminex Corporation's fluorescent analytical test instrumentation marketed under the name Luminex Instrument.

Full statement: https://www.luminexcorp.com/eu/end-user-terms-and-conditions/

Please note: The test is for research use only in the US – not for use in diagnostic procedures.

Genetic Analysis AS
Ulvenveien 80
0581 Oslo, Norway

**Document ID**	S8-044-A1
**Revision**	12
**Date of approval**	September 2024
**Available from**	https://ga-map.com/resources/

TABLE OF CONTENTS

PRODUCT INFORMATION 4

Definitions of symbols 4
Warnings and precautions 4
Principle of analysis 4
Intended use 4
Content of the reagent kit 5
Shelf life and storage of Reagent kit 5

GENERAL ASSAY INFORMATION 6

Procedure outline 6
Sample storage 6
Assay quality control scheme 7
Plate setup 7
General procedure notes 8
Materials required but not provided 9

EXTRACTION VALIDATION 9

Validation of new instruments and reagent kits for extraction 9

SAMPLE COLLECTION 10

Sampling procedure 10
Sample receipt at site of analysis 10

LABORATORY PROCEDURE 11

Step 1 - Genomic DNA extraction 11
Step 2 - Amplification of the bacterial 16S rRNA gene 13
Step 3 - Quantification of PCR product 14
Step 4 - Clean-up of PCR product 15
Step 5 - End-Labeling of Probe set 16
Step 6 – Hybridization and Signal detection 18

RESULT GENERATION AND INTERPRETATION 21

GA-map® Analyzer 21
Data QC and result generation 21
Interpretation of results 21

PERFORMANCE CHARACTERISTICS 22

Analytical performance characteristics 22
Diagnostic performance characteristics 22

LIMITATIONS 22

TROUBLESHOOTING 23

Too high/low DNA concentration for patient sample 23
Too high/low DNA concentration for control sample 23
Droplets observed on plate after scan 23
QC errors in the GA-map® Analyzer software 24

APPENDICES 25

DEFINITIONS OF SYMBOLS

Symbol	Definition	Symbol	Definition
	Manufacturer		Temperature storage limitation
LOT	Lot number		Expiry date
	Reference to electronic IFU	Σ	Number of tests
REF	Catalogue number		Procedure can be paused
IVD	*In vitro* diagnostic medical device	CE	Meets the applicable provision according to the EU Directive 98/79/EC on IVDs

WARNINGS AND PRECAUTIONS

Fecal samples should be treated as potentially infectious material and require the use of BSL-2 grade laboratory equipment and precautions. This involves the use of appropriate PPE, biological safety cabinet, proper waste disposal and risk-minimizing routines for sample handling.

Appropriate skin and eye protection should be worn during usage of the DNA isolation kit (mag™ maxi, LGC genomics). Do not use bleach for decontamination of liquid waste from the extraction process.

The operators must have general skills in molecular biology laboratory techniques to perform the GA-map® Dysbiosis Test Lx v2.

PRINCIPLE OF ANALYSIS

The GA-map® Dysbiosis Test Lx v2 is a diagnostic test that maps the intestinal microbiota profile for a selected set of bacteria and allows diagnosis of dysbiosis of patients in a clinical setting.

The GA-map® platform uses probes that target variable regions (V3 to V9) of the bacterial 16S rRNA gene to characterize and identify bacteria present (Casén C *et al*. (2015) Aliment Pharmacol Ther.). The targets are identified in a molecular multiplex assay that utilizes the Single Nucleotide Primer Extension (SNuPE) technology patented by Professor Knut Rudi (US6617138). A unique algorithm takes advantage of all the data generated by the detection of the SnuPE products to determine dysbiosis level in the sample. The algorithm is incorporated in the GA-map® Analyzer software that accompanies the test.

INTENDED USE

GA-map® Dysbiosis Test Lx v2 is intended to be used as a fecal gut microbiota DNA analysis tool to identify and characterize dysbiosis in adults.

Indications for use

- Determine if a patient is non-dysbiotic or dysbiotic (and degree of dysbiosis) according to cut-off given in the GA-map® Dysbiosis Test Lx v2.

In patients where organic disorders have been ruled out, the test can be used for identification of dysbiosis in:
    - IBS patients
    - IBD patients
    - symptomatic non-IBD patients (patients with negative colonoscopy results)
- Establish a gut microbiota profile given bacteria levels compared to a normobiotic reference in:
    - IBS patients
    - IBD patients
    - Symptomatic non-IBD patients (patients with negative colonoscopy results)
    - patients with functional gastrointestinal disorders
- Monitor dysbiosis during treatment of IBS or non – IBD or IBD.

CONTENT OF THE REAGENT KIT

The volumes provided in the GA-map® Dysbiosis Test Lx v2 kit are sufficient for 96 reactions, including controls. The reagents are provided in two boxes; Box A and Box B.

Part number	Component name	Volume (µl)
	**Box A**	
2205	GA-map® PCR MasterMix	2 × 1150
4005	GA-map® PCR polymerase	90
6005	GA-map® End-Labeling polymerase	75
2204	GA-map® End-Labeling MasterMix	2 × 1090
2405	GA-map® Biotin	140
1102	GA-map® End-Labeling ctrl pos (QCC30)	65
2452	GA-map® End-Labeling ctrl neg (QCC29)	65
1304	GA-map® Hybridization ctrl (HYC01)	820
1402	GA-map® Kit ctrl pos (QCC23)	30
1502	GA-map® Kit ctrl neg (QCC33)	30
2404	GA-map® rSAP	250
	**Box B**	
9003	GA-map® Bead set	84
2305	GA-map® SAPE	150
2426	GA-map® Hybridization Buffer	14000
2427	GA-map® Detection Buffer	14000

SHELF LIFE AND STORAGE OF REAGENT KIT

The shelf life of the reagent kit is set to 18 months from date of production. The expiry date is indicated on the box label and the content should not be used after the expiry date. The kit can be reused maximum five times.

Kit box	Storage temperature	Maximum freeze-thaw cycles
GA-map® Dysbiosis Test Lx v2, Box A	<-15°C	5*
GA-map® Dysbiosis Test Lx v2, Box B**	2-8°C	NA

* The GA-map® PCR MasterMix and End-Labeling MasterMix are provided in two aliquots with three freeze-thaw cycles.
** The GA-map® Hybridization buffer and Detection buffer should be equilibrated to ambient temperature before use.

GENERAL ASSAY INFORMATION

PROCEDURE OUTLINE

The GA-map® Dysbiosis Test Lx v2 lab procedure is divided into six main steps, which are described in more detail in the Lab Procedure section. The outline and instructions must be followed carefully.

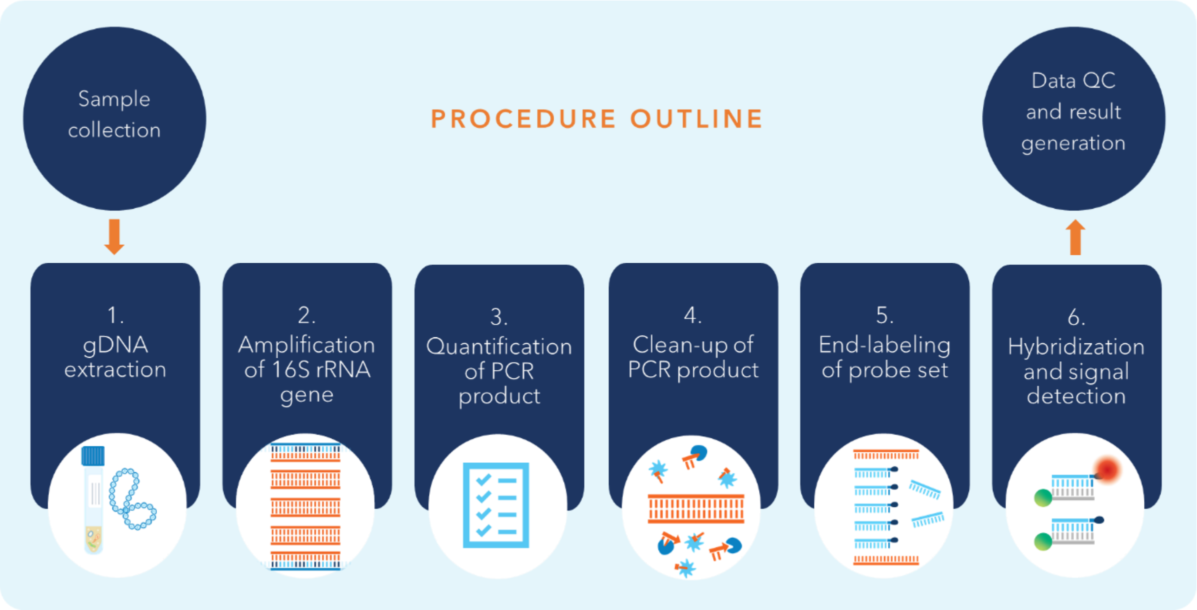

SAMPLE STORAGE

The recommended storage conditions for fecal samples collected on Copan eNAT™ buffer.

Sample material	Storage temperature	Maximum storage time	Maximum freeze-thaw cycles
Fecal sample on eNAT™ buffer	RT (<40°C)*	14 days	NA
	2-8°C**	4 weeks	NA
	<-15°C	Prolonged storage	2

* Samples on eNAT™ buffer should be kept at +4°C after reception.
**For storage >4 weeks, the tubes should be frozen ≤-15°C.

The sample intermediates are products of the different steps in the lab procedure. The procedure can be paused at several different steps, as indicated by the maximum storage time of the sample

intermediates. The procedure description also contains several "procedure can be paused" symbols, which indicate possibility for overnight pauses.

Sample intermediate	Storage temperature	Maximum storage time	Maximum freeze-thaw cycles
gDNA undiluted and 1:50 dilution	2-8°C	3 weeks	NA
	<-15°C	Prolonged storage	9
16S rRNA PCR product	2-8°C	48 hours	NA
16S rRNA PCR product, rSAP-treated	2-8°C	7 days	NA
End-Labeling product	2-8°C	2.5 hours	NA
	<-15°C	2 weeks	1
Hybridization product	RT, protected from light	24 hours (Luminex® 200™)	NA
		4 hours (MAGPIX®)	NA

ASSAY QUALITY CONTROL SCHEME

An overview of the different controls included in the assay (both required and optional) is given in the table below.

Control name	Control ID	Comment	Included from step
Extraction control negative	QCC02	Lysis Buffer BLM from DNA extraction reagent kit	Step 1 – Genomic DNA extraction
GA-map® Kit ctrl pos	QCC23	Included in kit	Step 2 – Amplification of the bacterial 16S rRNA gene
GA-map® Kit ctrl neg	QCC33	Included in kit	Step 2 – Amplification of the bacterial 16S rRNA gene
PCR control negative	QCC05	The water used to dilute gDNA	Step 2 – Amplification of the bacterial 16S rRNA gene
GA-map® End-Labeling ctrl neg	QCC29	Included in kit	Step 5 – End-Labeling of probe set
GA-map® End-Labeling ctrl pos	QCC30	Included in kit	Step 5 – End-Labeling of probe set
GA-map® Hybridization ctrl	HYC01	Included in kit	Step 6 – Hybridization and signal detection

PLATE SETUP

The figures below illustrate the recommended arrangement of assay controls in the 96-well plates. The patient samples can be placed in any other well on the plate. All steps should be performed by working in columns, from column 1 to column 12, and an 8-channel pipette should be used whenever applicable. The naming of assay controls is especially important during step 6 of the assay, as the result-generating software requires these controls to perform data QC.

Step 1 - Genomic DNA extraction

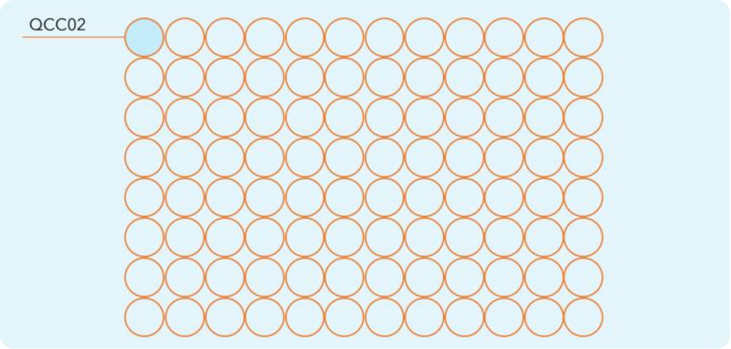

Step 2 - Amplification of the bacterial 16S rRNA

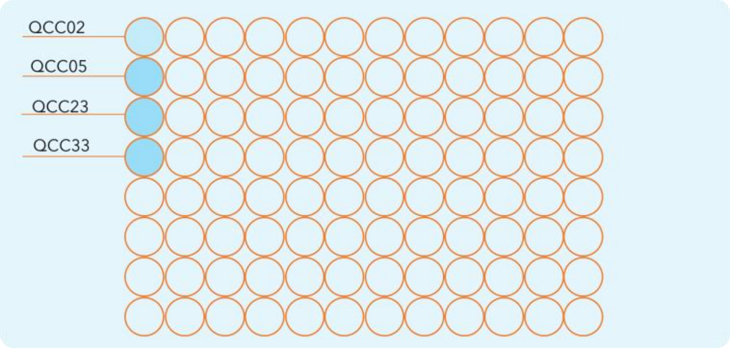

Step 5 - End-Labeling of Probe set and Step 6 - Hybridization and signal detection

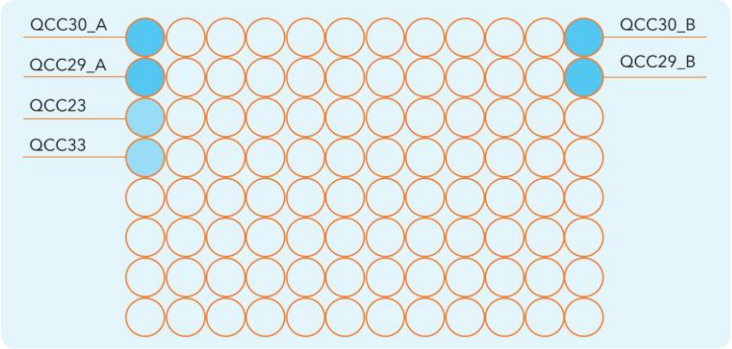

GENERAL PROCEDURE NOTES

- Before start, it is recommended that the user confirms that required equipment, materials and reagents are available for each step of the procedure.
- A plate setup with controls and samples should be prepared for each step of the procedure and reagent volumes should be calculated according to the sample number.
- Appendix 4 – GA-map® Dysbiosis Test Lx v2 Run log (available upon request) can be used for plate setup and volume calculation.
- Components from different lots of the GA-map® Dysbiosis Test Lx v2 kit should not be mixed.
- Reagents from Box A, except for enzymes, should be thawed on ice/cooling block before use and kept on ice/cooling block during handling.
- All enzymes should be kept on a freezing block during handling. Due to high viscosity, the enzymes should be briefly spun down (not vortexed) before use, then slowly aspirated without pipetting up and down.
- The buffers from Box B should be kept at ambient temperature (15 to 25°C).

- Before use, all reagent tubes except enzymes should be vortexed at 2800 rpm for 3 seconds and briefly spun down.
- For all centrifugation steps (tubes and plates), unless otherwise specified, use a brief spin down up to 100 x g.
- In steps 2-5, the 96-well plates and reagents should be kept on ice/cooling block.
- In step 6, the 96-well plate and reagents except for the GA-map® Hybridization ctrl (HYC01) should be kept at ambient temperatures.

MATERIALS REQUIRED BUT NOT PROVIDED

Refer to Appendix 6 Installation Guide for a detailed list of required equipment, materials, and reagents. The lab should be equipped as recommended in the Installation Guide.

EXTRACTION VALIDATION

VALIDATION OF NEW INSTRUMENTS AND REAGENT KITS FOR EXTRACTION

The DNA extraction method described in the IFU is validated for use with the GA-map® Dysbiosis Test Lx v2. For other validated DNA extraction alternatives, refer to Appendix 7 GA-map® Dysbiosis Test Lx v2 Validated Equipment (available upon request).

In order to validate a new extraction procedure, a selection of samples with varying DI-scores must be extracted at both sites and analyzed together. Contact your local distributor or Genetic Analysis to order an extraction validation package.

SAMPLING PROCEDURE

The recommended sampling procedure with Copan eNAT™ tubes is described below. “Dry” samples (without buffer) can also be used. Contact Genetic Analysis for information on this. Other types of transport buffers/reagents may lead to a bias in the results and are not validated for use with the GA-map® Dysbiosis Test Lx v2. For validation of sampling methods, please contact Genetic Analysis for more information.

Materials:

- eNAT™ tube w/ regular FLOQSwab from Copan, cat no: 608CS01R (1ml) or 606CS01R (2ml)
- Fecal sampling container, e.g. sampling tray or Med Auxil sampling paper (sterile/clean).

Procedure:

1. Sampling can be performed at any convenient place, at any time of the day.
2. Sampling shall be done from one single bowel movement.
3. Collect the fecal sample in a collecting unit. Do not mix urine with the fecal sample, and never collect the sample directly from the toilet.
4. Open the package containing sample tube and swab and remove the swab stick holding above the red line to avoid contamination.
5. Dip the swab into the sample and roll until the swab is saturated with a thin layer of material. Do not over-saturate the swab! Only a small amount of sample material is needed for analysis.
6. Place the swab inside the sample tube and break off the top part of the stick (above the red line). Securely close the tube by tightening the cap and shake until the sample appears homogenous.
7. Mark the tube with name/ID and sampling date.
8. Secure the sample by placing the tube into a secondary container (tube or bag with absorbing unit).
9. Place the secured sample in the addressed envelope/bag/box and ship the sample as directed.

SAMPLE RECEIPT AT SITE OF ANALYSIS

Upon receipt of fecal samples, the shipment should be checked for leakage from the tubes or damage, and visually assessed for presence of mucus and blood.

Samples collected on eNAT™ buffer should be stored at 2-8°C or at ambient temperatures (<40°C) until analysis. If the time from sampling to analysis exceeds the recommended time frames (see the Sample Storage section), the sample tube should be frozen at ≤-15°C upon receipt.

STEP 1 - GENOMIC DNA EXTRACTION

Equipment, materials, and reagents for extraction with mag™ maxi DNA purification kit (LGC genomics)

No reagents from the GA-map® Dysbiosis Test Lx v2 kit are required for this step. Refer to Appendix 6 Installation Guide for specifications for equipment, materials, and reagents required in this step, in addition to workstation setup. Disposable materials and reagents required are also listed below.

Disposable materials required:

- Lysing Matrix-E tubes
- Tube for Lysis mix
- Plastic for the DNA extraction robot
- Adhesive PCR plate seal
- Microtiter sealing tape
- Microtiter plate w/ seal for gDNA dilution
- Reagent reservoir

Reagents required:

- Extraction control negative (Lysis Buffer BLM from DNA extraction reagent kit)
- DNA extraction reagent kit, including ethanol and acetone
- Water for dilution of gDNA

Preparing the sample and reagents for gDNA extraction

Note: Protease from LGC DNA isolation kit must be prepared in accordance with LGC DNA isolation kit instruction before use. Thaw on ice before use. The BLM2 buffer must be prepared by adding acetone in accordance with LGC DNA isolation kit instruction before use.

1. If frozen, samples on Copan eNAT™ tubes should be thawed for ≥15 minutes.
2. Vortex the sample tubes to make feces homogeneous.
3. If using 1ml Copan eNAT™ tubes: Add 200µl Elution buffer to the Lysing Matrix-E tubes and then transfer 400µl sample from the Copan tubes to the Lysing Matrix-E tubes, using a pipette with wide orifice tips. Use the decapper for opening and closing of the Matrix-E tube screw caps.
   OR:
   If using 2ml Copan eNAT™ tubes: Transfer 600µl sample from the Copan tubes to the Lysing Matrix-E tubes, using a pipette with wide orifice tips. Use the decapper for opening and closing of the Matrix-E tube screw caps.

Performing gDNA extraction

1. Process the Lysing Matrix-E tubes with samples twice in the bead beater at 1800 rpm for 40 seconds with a 40 second pause between runs. Ensure that the racks are balanced.
2. Centrifuge samples at 1300 x g for 5 minutes in the plate centrifuge for Lysing Matrix-E tubes.
3. Prepare Lysis mix according to table below. Mix by pipetting.

Note: The negative extraction control is included from the lysis step. Remember to prepare Lysis protease mix also for this control.

Component in Lysing mix	µl per well
Lysis buffer BLM (LGC Genomics)	250
Protease (LGC Genomics)	20
**Total volume**	**270**

4. Transfer 270µl Lysis mix to all wells of a deep well plate according to plate setup.
5. Transfer 250µl sample supernatant from the centrifuged samples to the wells with Lysis mix, using a pipette with wide orifice tips. Use the decapper for opening and closing of the Matrix-E tube screw caps. Remember to add 250µl Lysis buffer BLM as negative extraction control. Mix well by pipetting.
6. Cover the plate with adhesive PCR plate seal. Make sure the plate is thoroughly sealed.
7. Carefully place the deep well plate to float in the water bath and incubate at 65°C for 15 minutes.
8. During the incubation, finalize preparation of buffer plates according to the table below and the plate setup. Seal the plates with microtiter sealing tape until use.

Plate Type	Plate #	Content	Volume
Deep well plate	1	MagMAX particles (vortex thoroughly before use)	20µl
		Ethanol	200µl
	2	Washing buffer BLM 1	720µl
	3	Washing buffer BLM 2	720µl
	4	Washing buffer BLM 2	720µl
Elution plate	5	Elution buffer BLM	200µl
Elution plate	6	Tip comb placed in KF microplate	N/A

9. Remove the deep well plate from the water bath. If any drop of buffer is visible under the plate seal after incubation, pulse spin before removing the film.
10. Add 400µl treated sample to plate#1 according to plate setup, using a pipette with wide orifice tips. Mix well by pipetting.
11. Turn on the DNA extraction robot, find the GA-map® program[1] and press Start. The robot will request plate#6 first, then plate#5 and so on. Unseal the plates before placing them in the machine. The procedure will take approximately 40 minutes.
12. When completed, remove plate#5 (elution plate). Seal the plate with microtiter sealing tape and centrifuge the plate for 1 minute at 1000 x g. Place the plate on ice/cooling block.
13. Discard plates #1, #2, #3, #4, and #6.
14. From plate#5, transfer 100µl of the eluted gDNA (avoid transferring the brown debris in the bottom) into a 96-well microtiter plate.
15. Dilute the gDNA 1:50 by mixing 5µl gDNA and 245µl nuclease free water. Mix well.

If pausing at this step, the gDNA can be stored at 2-8℃ for up to 3 weeks (or alternatively at ≤-20℃ for long term storage).

[1] See chapter Appendice for details. Available upon request.

STEP 2 - AMPLIFICATION OF THE BACTERIAL 16S RRNA GENE

Equipment, materials, and reagents

GA-map® Dysbiosis Test Lx v2 kit reagents required for this step are listed in the table below.

GA-map® reagent	Storage	Handling method
GA-map® PCR MasterMix	GA-map® Dysbiosis Test Lx v2, Box A	Thaw on ice before use, keep on ice during handling.
GA-map® Kit ctrl pos (QCC23)		
GA-map® Kit ctrl neg (QCC33)		
GA-map® PCR polymerase	GA-map® Dysbiosis Test Lx v2, Box A	Keep on a freezing block during handling.

Refer to Appendix 6 Installation Guide for specifications for equipment, materials, and reagents required in this step, in addition to workstation setup. Disposable materials and reagents required are also listed below.

Disposable materials and reagents required:

- Microcentrifuge tube for PCR mix
- Microtiter plate for PCR with suitable lids or seal
- Microtiter sealing tape
- PCR control negative (same water as used for gDNA dilution in Step 1)

Performing the amplification of bacterial 16S rRNA gene

1. Keep thawed reagents and the diluted gDNA on ice/cooling block.
2. Prepare the PCR mix according to the table below. Mix by vortexing and briefly spin down.

**Component in PCR mix**	**µl per well**
GA-map® PCR MasterMix	19.25
GA-map® PCR polymerase	0.75
**Total volume**	**20.0**

3. Dispense 20µl of the PCR mix into the microtiter plate according to plate setup. Cover plate with microtiter sealing tape if relocating to a different room. Keep on ice/cooling block.
4. Add samples and controls to the PCR plate according to plate setup:
   - 5µl of the 1:50 diluted gDNA (samples and extraction control)
   - 5µl of the kit controls and PCR control negative
5. Mix well by pipetting.
6. Seal the plate.
7. The plate can be vortexed to ensure proper mixing.
8. Briefly pulse spin the plate up to 100 x g.
9. Initiate the PCR-program and load the plate on the thermal cycler.

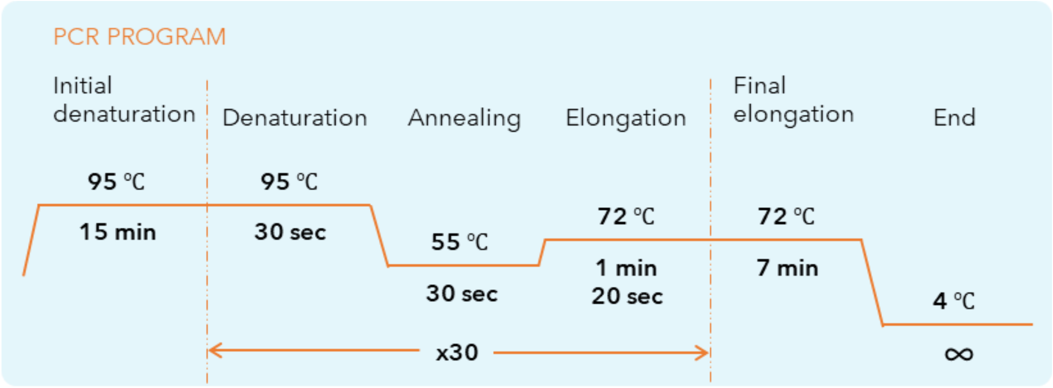

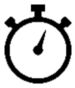

If pausing at this step, the PCR-product can be stored at 2-8°C for up to 48 hours.

STEP 3 - QUANTIFICATION OF PCR PRODUCT

The quality of the PCR product must be assessed to ensure optimal performance in further downstream processes. This quality control step is important for the functionality of the GA-map® Dysbiosis Test Lx v2. The following method for dsDNA quantification is recommended. Other systems must be validated for use with the GA-map® Dysbiosis Test Lx v2.

Equipment, materials, and reagents

Refer to Appendix 6 Installation Guide for specifications for equipment, materials, and reagents required in this step, in addition to workstation setup. Disposable materials and reagents required are also listed below.

Disposable materials and reagents required:

- Reagent reservoir
- Tubes/plates for DNA quantification
- Assay kit for DNA quantification

Performing the quantification of PCR product

1. Vortex and briefly spin down the 96-well plate containing 16S rRNA PCR product.
2. Keep PCR product on ice/cooling block.
3. Perform the quantification of PCR product according to applicable assay User Guide.
    a. Use 10µl of each standard
    b. Use 2µl of the PCR product for each sample
4. Perform QC according to the requirements described below.

QC requirements

The PCR yield should be within the concentration limits presented below.

Sample type	Lower limit	Upper limit
Patient samples and kit controls (QCC23 and QCC33)	≥17ng/µl	-
Negative controls (QCC02 and QCC05)	-	<5ng/µl

See Troubleshooting section if PCR product is confirmed to be outside the lower or upper limits.

STEP 4 - CLEAN-UP OF PCR PRODUCT

Equipment, materials, and reagents

GA-map® Dysbiosis Test Lx v2 kit reagents required for this step are listed in the table below.

GA-map® reagent	Storage	Handling method
GA-map® rSAP	GA-map® Dysbiosis Test Lx v2, Box A	Keep on a freezing block

Refer to Appendix 6 Installation Guide for specifications for equipment, materials, and reagents required in this step, in addition to workstation setup. Disposable materials and reagents required are also listed below.

Disposable materials and reagents required:

- Microcentrifuge tube for rSAP mix
- PCR grade water
- Reagent reservoir

Performing clean-up of PCR product

1. Vortex and briefly spin down the 96-well plate containing 16S rRNA PCR product.
2. Keep reagents and the PCR product on ice/cooling block.
3. Prepare rSAP mix according to the table below. Mix by pipetting and briefly spin down.

Component in rSAP mix	µl per µl PCR product	Example: µl per well when using 23µl PCR product
GA-map® rSAP	0.1	2.3
PCR grade water	0.3	6.9
**Total volume**	**0.4**	**9.2**

4. Transfer the rSAP mix to a reagent reservoir.
5. Add the appropriate volume to the PCR products, mix well by pipetting. The QCC02 and QCC05 controls are not required for the downstream steps, and do not need rSAP mix.
6. Seal the plate.

Note: Due to the viscosity of the rSAP enzymes, additional mixing of the plate using a vortexer is recommended. Insufficient mixing may lead to a bias in the results.

7. Briefly pulse spin the plate to 100 x g.
8. Initiate the clean-up program and load the plate on the thermal cycler.

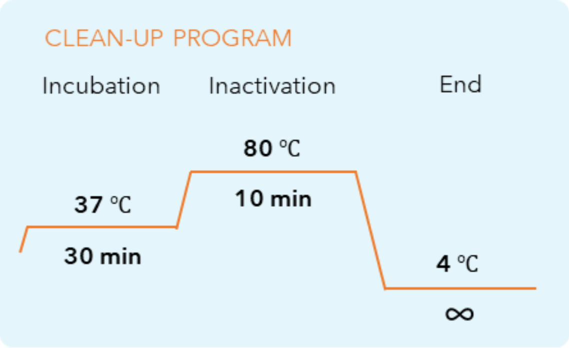

If pausing at this step, the rSAP-treated PCR product can be stored at 2-8°C for up to 7 days.

STEP 5 - END-LABELING OF PROBE SET

Equipment, materials, and reagents

GA-map® Dysbiosis Test Lx v2 kit reagents required for this step are listed in the table below.

GA-map® reagent	Storage	Handling method
GA-map® End-Labeling MasterMix	GA-map® Dysbiosis Test Lx v2, Box A	Thaw on ice before use, keep on ice during handling
GA-map® Biotin		
GA-map® End-Labeling ctrl pos (QCC30)		
GA-map® End-Labeling ctrl neg (QCC29)		
GA-map® End-Labeling polymerase	GA-map® Dysbiosis Test Lx v2, Box A	Keep on a freezing block during handling

Refer to Appendix 6 Installation Guide for specifications for equipment, materials, and reagents required in this step, in addition to workstation setup. Disposable materials and reagents required are also listed below.

Disposable materials and reagents required:

- Microcentrifuge tube for End-labeling mix
- New microtiter plate for End-labeling with suitable lids or seal
- Microtiter sealing tape

Performing End-Labeling of probe set

1. Vortex and briefly spin down the 96-well plate containing rSAP-treated PCR product.
2. Keep thawed reagents and the rSAP-treated PCR product on ice/cooling block.
3. Prepare End-Labeling mix according to the table below. Mix by vortexing and briefly spin down.

**Component in End-Labeling mix**	**µl per well**
GA-map® End-Labeling MasterMix	18.125
GA-map® Biotin	1.25
GA-map® End-Labeling polymerase	0.625
**Total volume**	**20**

4. Dispense 20µl of the End-Labeling mix into a microtiter plate according to plate setup. Cover plate with microtiter sealing tape if relocating to a different room. Keep on ice/cooling block.
5. Add samples and controls to the End-Labeling plate according to plate setup:
   - 5µl of the rSAP-treated PCR samples and kit controls
   - 5µl of the End-Labeling controls
6. Mix well by pipetting.
7. Seal the plate.
8. Briefly pulse spin the plate to 100 x g.
9. Initiate the End-Labeling program and load the plate on the thermal cycler.

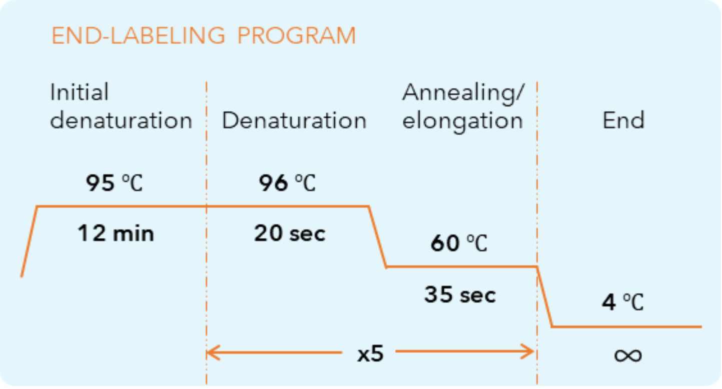

10. While the samples are in the thermal cycler, proceed to preparation for Step 6 – Hybridization and Signal detection.
11. Directly after completion of the End-Labeling program, transfer the PCR plate from the cycler to a freezing block.

NOTE: The End-Labeling product is labile. To prevent unspecific signals, keep the End-Labeling product on a freezing block during handling and storage. Ensure that the time between End-Labeling (this step) and Hybridization (next step) is less than 2.5 hours.

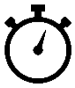

Alternatively, if stopping at this step, the End-labeling product can be stored at -20°C for up to two weeks.

STEP 6 – HYBRIDIZATION AND SIGNAL DETECTION

Equipment, materials, and reagents

GA-map® Dysbiosis Test Lx v2 kit reagents required for this step are listed in the table below.

GA-map® reagent	Storage	Handling method
GA-map® Hybridization ctrl (HYC01)	GA-map® Dysbiosis Test Lx v2, Box A	Thaw on ice before use, keep on ice during handling
GA-map® Bead set	GA-map® Dysbiosis Test Lx v2, Box B	Ambient temperature (15-25°C) during handling
GA-map® SAPE		
GA-map® Hybridization Buffer	Ambient temperature (15-25°C)	Ambient temperature (15-25°C)
GA-map® Detection Buffer		

Refer to Appendix 6 Installation Guide for specifications for equipment, materials, and reagents required in this step, in addition to workstation setup. Disposable materials and reagents required are also listed below.

Disposable materials and reagents required:

- Microcentrifuge tubes for Hybridization bead mix and Reporter mix
- Hybridization plate
- Two sealing films for Hybridization plate
- Reagent reservoir

Performing Hybridization and SAPE-labeling

NOTE! All reagents required for this step (except for the GA-map® Hybridization ctrl) are to be kept at ambient temperature. The hybridization plate should also be kept at ambient temperature during preparation and during the buffer exchange steps. Lower temperatures may lead to a drop in signal intensities.

1. Make sure the Luminex detection instrument is calibrated and ready for use.
2. Vortex and briefly spin down the 96-well plate containing End-Labeling product. Keep on ice block.
3. Prepare the Hybridization bead mix according to the table below.

Note! The GA-map® Bead set must be resuspended by vortexing for ≥10 seconds.

**Component in Hybridization bead mix**	**µl per well**
GA-map® Hybridization Buffer	32.2
GA-map® Hybridization ctrl (HYC01)	7
GA-map® Bead set	0.8
**Total volume**	**40**

4. Resuspend the prepared Hybridization bead mix by vortexing for 10 seconds.
5. Dispense 40µl of the Hybridization bead mix in Hybridization plate according to plate setup. Vortex the Hybridization bead mix thoroughly between each 24 wells.

6. Transfer 10µl of the End-Labeling product to the Hybridization plate according to plate setup. Mix well by pipetting.

Note: Due to the different salt concentrations of the reaction components, additional mixing is recommended. Mix by pipetting up and down minimum 6 times upon distribution of the End-Labeling product. Insufficient mixing may lead to a bias in the results.

7. Cover the plate with a sealing film for Hybridization plate.
8. Initiate the Hybridization program and load the plate into the thermal cycler. Set a timer to 18 minutes**.**

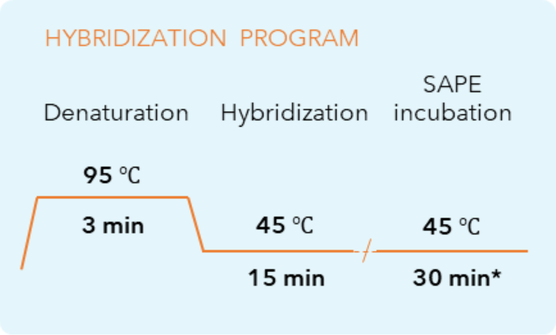

*Program the instrument for ∞ time at SAPE incubation stage.

9. Prepare the Reporter mix according to the table below.

Component in Reporter mix	µl per well
GA-map® Hybridization Buffer	23.8
GA-map® SAPE	1.2
**Total volume**	**25**

10. After completion of the 18 minutes (Denaturation and Hybridization stages of the thermal cycler program), keep the plate at 45°C in the thermal cycler and add 25 µl of the Reporter mix to each well. Mix gently by pipetting.
11. Cover the plate with a new sealing film for Hybridization plate, close the lid and leave the program running for an additional 30 minutes (SAPE incubation stage of the thermal cycler program). Use a timer.

Preparation for signal detection

1. While the plate is incubating, prepare for scan on the Luminex detection instrument:
   a. In xPONENT, create a new batch from the correct protocol, depending on the type of Luminex instrument:

      Luminex® 200™: "GA-map Dysb. Test Lx v2" version 1.1 (Appendix 3, available upon request)
      MAGPIX®: "GA-map Dysb. Test Lx v2_MP" version 1.0 (Appendix 5, available upon request)

   b. Give the batch a unique file name including the GA-map® Dysbiosis Test Lx v2 reagent kit lot number and run date. The correct kit lot is needed for processing using the GA-map® Analyzer software.

c. Import sample and control IDs to the respective wells and optional additional information. Make sure that each sample ID is unique and traceable in your system.
d. Note that some wells in the first and last column are reserved for controls: ensure the names of the control IDs are correct.
e. Make sure the sample and control IDs correspond to the correct positions on the plate.

Note! The buffer exchange is a time sensitive step. Limit the time the wells are left without buffer to ≤3 minutes. Beads drying out may lead to signal loss!

2. When the 30 minutes SAPE incubation stage has completed, take the plate out of the thermal cycler and place it on a ring magnet plate for 60 seconds.
3. After the beads have collected on the side of the wells, carefully remove all the hybridization/ reporter solution.
4. Remove the plate from the ring magnet plate and add 75µl of GA-map® Detection Buffer to each well. Mix by pipetting until all beads are resuspended (approximately 10 times).

Note! Insufficient resuspension after buffer exchange may lead to low bead count errors. Perform a visual check of the plate to ensure the beads are properly resuspended. In case of a visible band of beads across the wells, repeat the resuspension process in step 4 above.

Prior to scan, the Hybridization-product can be stored at ambient temperature in the dark (resuspend the beads by pipetting up and down a few times before continuing the process):

Luminex® 200™: plate can be stored for up to 24 hours prior to scan
MAGPIX®: plate can be stored for up to 4 hours prior to scan

5. Move the plate to the Luminex detection instrument.
6. Click the Run button to start analysis and confirm by pressing OK.
7. When the run is complete, export the results in a .csv format:
    a. From the Results > Saved Batches tab, select the batch and click Exp Results.
    b. Save file in the comma separated values (.csv) format. The file can be processed in the GA-map® Analyzer software for quality check and patient result generation. Do not open the file in Excel before analysis, as this may corrupt the file!
8. Remove the plate and discard it according to local regulations.

GA-MAP® ANALYZER

The GA-map® Analyzer, part no. 2801, allows quality check and patient sample result generation from data generated with the GA-map® Dysbiosis Test Lx v2 assay on a Luminex signal detection instrument. Refer to the GA-map® Analyzer Software Manual for use of the software.

DATA QC AND RESULT GENERATION

The GA-map® Analyzer software performs the following tasks:

1. **Plate QC check**: The GA-map® Analyzer software checks that all the required quality controls are present in the uploaded csv file and that the plate QC parameters are met. It is therefore important to use the controls and names specified in the Plate setup section. If the plate QC check does not pass, the plate, including all samples, is invalid.

2. **Sample QC check**: If the plate is found valid, the individual samples are evaluated. Samples that do not meet the raw data signaling level QC parameters will be flagged as invalid.

3. **Data normalization and result generation for each sample:**
    a. DI score
    b. Bacteria Abundance Scores
    c. Bacteria profiles

4. **Report generation**: Patient reports in the form of an xml-file can be generated.

Please refer to the GA-map® Analyzer Software Manual for detailed instructions on how to use the software and generate reports.

INTERPRETATION OF RESULTS

A report template in a suitable format is required for presentation of the data in the xml-file. The xml-file contains information which in a readable report can be presented in the following three levels:

Dysbiosis Index scale: To determine the degree of dysbiosis compared to a normal healthy reference population, the test result is given as a Dysbiosis Index score (DI) consisting of five levels ranging from 1 to 5, where 1 and 2 are non- dysbiotic and 3 to 5 are dysbiotic. DI score 3 is considered mildly dysbiotic, while scores 4 to 5 are severely dysbiotic. The DI scale is CE-marked.

Bacteria Profiles: Five different functional profiles, as well as bacteria diversity, are reported, each representing a set of unique bacteria signatures. The profiles are reported as either Balance or Imbalance followed by a comment. The bacteria diversity is reported as either Low or Expected.

Bacteria Abundance Table: The Bacteria Abundance Score of 48 preselected bacteria markers relative to the value of a normal healthy reference population. The relative abundance of each bacteria marker can be normal, reduced, or elevated.

PERFORMANCE CHARACTERISTICS

ANALYTICAL PERFORMANCE CHARACTERISTICS

Precision: Precision analysis for GA-map® Dysbiosis Test Lx v2 show acceptable levels of repeatability and reproducibility. All samples had a standard deviation ≤0.2 for DI score.

DIAGNOSTIC PERFORMANCE CHARACTERISTICS

An independent validation study has been performed to investigate the classification performance of GA-map® Dysbiosis Test Lx v2. The validation study comprised a total of 213 IBD and non-IBD samples from fecal sample recruitment hospitals in Norway, Sweden and Denmark. A separate clinical validation on a healthy normal cohort collected in Germany was performed using 54 fecal samples. All samples were from unique feces donors not included in previous development of GA-map® Dysbiosis Test Lx v2.

The performance characteristics (distribution of normobiotic and dysbiotic per patient category) are shown below.

	IBD	non-IBD	Healthy normal
No of samples DI>2	87	65	9
No of samples DI<2	33	28	45
Total no of samples	120	93	54
% DI>2	73	70	17
% DI<2	28	30	83

LIMITATIONS

- GA-map® Dysbiosis Test Lx v2 has not been documented for use to discriminate between IBS, IBD, and other types of gastro-intestinal disorders.
- GA-map® Dysbiosis Test Lx v2 is intended for use with patients at the age between 18 and 70 years.
- Repeated freeze-thaw cycles of the fecal sample may change microbiota composition.
- The analysis method, including the sample collection and extraction method, must be performed as described to generate valid GA-map® Dysbiosis Test Lx v2 results. Alternative methods require validation.

TOO LOW DNA CONCENTRATION FOR PATIENT SAMPLE

If the PCR quantification results for a single patient sample is outside the accepted range, the sample in question must be re-analyzed, either from PCR through preparing a new dilution or performing a new extraction. In case of repeated failure for the same sample upon re-extraction, the patient must be asked to donate a new sample.

TOO HIGH/LOW DNA CONCENTRATION FOR CONTROL SAMPLE

If the PCR quantification results for GA-map® Kit ctrl pos (QCC23), the GA-map® Kit ctrl neg (QCC33) and/or the PCR control negative (QCC05) are outside the accepted range, the PCR setup should be repeated from the amplification step. A failed kit control suggests a failure in the amplification itself (e.g. PCR master mix or temperature cycling), not with the extracted gDNA samples.

If the PCR quantification results for extraction control (QCC02) is outside the accepted range, the analysis should be repeated from genomic gDNA extraction.

DROPLETS OBSERVED ON PLATE AFTER SCAN

If observed splash between wells during the plate scan, a re-run from step 5 End-labeling or step 6 Hybridization if the End-labeling product is still valid is recommended to exclude possibility for sample contamination. If the splash between wells during the scan persists, perform following actions:

1. Perform Enhanced Startup Routine and follow the instructions in the xPONENT software.
2. Rinse the sample probe according to the weekly maintenance routine “Clean the sample probe” in the Luminex instrument user manual.
3. Change the sample probe if the probe rinse does not stop the spillage issue.
4. Cover the plate with a Pierceable foil for Hybridization plate to prevent spillage during plate scan if step 1 and 2 does not help and the splashing persists during plate scan. Contact GA support for recommendations of foil.

QC ERRORS IN THE GA-MAP® ANALYZER SOFTWARE

Any of the following error messages might occur when analyzing the data in the GA-map® Analyzer.

Error message	Interpretation	Possible causes	Suggested action
Kit control #1/ Kit control #2/<br>*End-labeling ctrl positive* not found on plate	QCC23/ QCC33/ QCC30 controls are not detected on the plate.	Incorrect naming of the Control IDs in the .csv file; see assay quality control scheme and plate setup for correct naming.<br><br>Controls are omitted from analysis or sample list; see assay quality control scheme and plate setup for correct control scheme.	Open the .csv file as a .txt file and manually correct the naming<br>OR<br>Locate the run under "Saved Batches" in the xPONENT software and select "Replay batch" using correct naming.<br><br>If one or more controls were not included in the analysis, repeat the analysis with the correct controls.
Kit control #1 and/or *Kit control #2* profile error	QCC23 and/or QCC33 controls have a DI score outside the accepted range.	Error in the 16S PCR or subsequent steps of the analysis.	Repeat the procedure from Step 2 (Amplification of the bacterial 16S rRNA gene) for all samples and controls.
End-labeling ctrl positive total signal above/below the limit	QCC30 total signal too high/low.	Error in the End-Labeling or subsequent steps of the analysis.	Repeat the procedure from Step 5 (End-Labeling of Probe set) for all samples and controls.
End-labeling ctrl negative total signal above the limit	QCC29, background signals are too high.	Error in the End-Labeling or subsequent steps of the analysis.	Repeat the procedure from Step 5 (End-Labeling of Probe set) for all samples and controls.
Hybridization error for […]	HYC01 has too high/low signal in the affected sample(s).	Error in the Hybridization step or subsequent steps of the analysis.	Repeat the procedure from Step 5 (End-Labeling of Probe set) or Step 6 (Hybridization and signal detection) for the affected samples and all controls.
Universal target error for […]	UNI05 has too low signal in the affected sample(s).	Error in the Step 2 (Amplification of the bacterial 16S rRNA gene) or subsequent steps of the analysis.	Repeat the procedure from Step 2 (Amplification of the bacterial 16S rRNA gene) for affected samples and all controls.
Low bead count for […]	One or more of the probes for given sample have bead count below the lower limit.	Too little of the GA-map® Bead set added during preparation of the Hybridization plate.<br><br>Loss of beads or incomplete resuspension of beads during the buffer exchanging step.	Repeat the procedure from Step 5 (End-Labeling of Probe set) or Step 6 (Hybridization and signal detection) for the affected samples and all controls.

Appendix 1: GAmap_v1.kf2 (For MagMAX™Express-96 instrument)

Appendix 2: GAmap_v2.bdz (For KingFisher Flex instrument)

Appendix 3: GA-map Dysb.Test Lx v2[1.1].lxt2

Appendix 4: GA-map Dysbiosis Test Lx v2 Run log.xlsm

Appendix 5: GA-map Dysb.Test Lx v2_MP[1.0].lxt2

Appendix 6: GA-map® Dysbiosis Test Lx v2 Installation Guide.pdf

Appendix 7: GA-map® Dysbiosis Test Lx v2 Validated Equipment.pdf

All appendices are available upon request from support@genetic-analysis.com

Genetic Analysis AS | Ulvenveien 80 | 0581 OSLO | Norway

www.genetic-analysis.com | Phone: +47 48 32 16 10 | E-mail: support@genetic-analysis.com

Registered names, trademarks, etc. used in this document, even when not specifically marked as such, are not to be considered unprotected by law. All trademarks and registered trademarks mentioned herein are the property of their respective owners.

© 2021-2024 Genetic Analysis, all rights reserved.
